# Investigations of Kidney Dysfunction-Related Gene Variants in Sickle Cell Disease Patients in Cameroon (Sub-Saharan Africa)

**DOI:** 10.3389/fgene.2021.595702

**Published:** 2021-03-15

**Authors:** Valentina J. Ngo-Bitoungui, Suzanne Belinga, Khuthala Mnika, Tshepiso Masekoameng, Victoria Nembaware, René G. Essomba, Francoise Ngo-Sack, Gordon Awandare, Gaston K. Mazandu, Ambroise Wonkam

**Affiliations:** ^1^West African Centre for Cell Biology of Infectious Pathogens, University of Ghana, Legon-Accra, Ghana; ^2^Division of Human Genetics, Department of Medicine, Faculty of Health Sciences, University of Cape Town, Cape Town, South Africa; ^3^Department of Microbiology Haematology and Immunology, University of Dschang, Yaoundé, Cameroon; ^4^Centre Pasteur of Cameroon, Yaoundé, Cameroon; ^5^National Public Health Laboratory, Yaoundé, Cameroon; ^6^Department of Microbiology, Parasitology, Haematology, Immunology and Infectious Diseases, Faculty of Medicine and Biomedical Sciences, University of Yaounde 1, Yaounde, Cameroon; ^7^Faculty of Medicine and Pharmaceutical Sciences, University of Douala, Douala, Cameroon; ^8^African Institute for Mathematical Sciences, Muizenberg, Cape Town, South Africa

**Keywords:** sickle cell disease, kidney dysfunctions, gene variants, cameroon, Africa

## Abstract

**Background:**

Renal dysfunctions are associated with increased morbidity and mortality in sickle cell disease (SCD). Early detection and subsequent management of SCD patients at risk for renal failure and dysfunctions are essential, however, predictors that can identify patients at risk of developing renal dysfunction are not fully understood.

**Methods:**

In this study, we have investigated the association of 31 known kidney dysfunctions-related variants detected in African Americans from multi-ethnic genome wide studies (GWAS) meta-analysis, to kidney-dysfunctions in a group of 413 Cameroonian patients with SCD. Systems level bioinformatics analyses were performed, employing protein-protein interaction networks to further interrogate the putative associations.

**Results:**

Up to 61% of these patients had micro-albuminuria, 2.4% proteinuria, 71% glomerular hyperfiltration, and 5.9% had renal failure. Six variants are significantly associated with the two quantifiable phenotypes of kidney dysfunction (eGFR and crude-albuminuria): *A1CF-rs10994860* (*P* = 0.02020), *SYPL2*-*rs12136063* (*P* = 0.04208), and *APOL1 (G1)-rs73885319* (*P* = 0.04610) are associated with eGFR; and *WNT7A-rs6795744* (*P* = 0.03730), *TMEM60-rs6465825* (*P* = 0.02340), and *APOL1 (G2)-rs71785313* (*P* = 0.03803) observed to be protective against micro-albuminuria. We identified a protein-protein interaction sub-network containing three of these gene variants: *APOL1, SYPL2*, and *WNT7A*, connected to the Nuclear factor NF-kappa-B p105 subunit (NFKB1), revealed to be essential and might indirectly influence extreme phenotypes. Interestingly, clinical variables, including body mass index (BMI), systolic blood pressure, vaso-occlusive crisis (VOC), and haemoglobin (Hb), explain better the kidney phenotypic variations in this SCD population.

**Conclusion:**

This study highlights a strong contribution of haematological indices (Hb level), anthropometric variables (BMI, blood pressure), and clinical events (i.e., vaso-occlusive crisis) to kidney dysfunctions in SCD, rather than known genetic factors. Only 6/31 characterised gene-variants are associated with kidney dysfunction phenotypes in SCD samples from Cameroon. The data reveal and emphasise the urgent need to extend GWAS studies in populations of African ancestries living in Africa, and particularly for kidney dysfunctions in SCD.

## Introduction

Sickle Cell Disease (SCD) is a monogenic disease with high prevalence and high mortality rates in Africa. Globally, SCD is estimated to affect more than 300,000 births per year, with nearly two-thirds occurring in sub-Sahara Africa (Piel et al., 2013). Cameroon is a sub-Saharan African country with an estimated SCD carrier frequency rate between 8 and 34% (Weatherall and Clegg, 2001). Although Cameroon declared SCD as a public health priority, access to care and treatment is still limited due to lack of a national medical insurance, leaving SCD patients to self-fund or depend on familial financial support. Therefore, medical care costs are often not met (Wonkam et al., 2014) and patients frequently suffer from severe SCD complications such as kidney dysfunction (Geard et al., 2017).

Renal failure caused by recurrent episodes of ischemia-reperfusion injury and haemolytic anaemia, occurs in 5–18% of SCD patients and is associated with an increased risk of early mortality (Platt et al., 1994; Gladwin, 2017). The prevention of renal failure relies on early detection and management of kidney dysfunction. In SCD patients, renal failure can be caused by gradual infiltration of glomerulus, which leads to glomerular sclerosis or promotes progression of micro-albuminuria to macro-albuminuria/proteinuria and finally to nephrotic-range proteinuria (Nath and Hebbel, 2015). Micro-albuminuria is prevalent in 26–68% of adult patients (Ataga et al., 2014; Gosmanova et al., 2014) and is the most sensitive early clinical marker for glomerular damage and other types of kidney dysfunction. Recent studies have demonstrated that the co-inheritance of alpha-thalassemia with SCD and/or specific variants in the HbF promoting loci can delay the clinical progression of kidney disease in African American SCD patients (Saraf et al., 2017). In addition, genetic variations in two coding regions of *Apolipoprotein L1 (APOL1)* and *Heme oxygenase 1 (HMOX1)* genes have been associated to chronic kidney disease (Genovese et al., 2010; Tzur et al., 2010), and to SCD nephropathy (Saraf et al., 2015; Schaefer et al., 2016).

In a previous study, we showed that variants in *APOL1* and *HMOX1* variants are associated with kidney dysfunctions using a targeted SNP based approach. Further investigations reveal that these variants are associated with albumin creatinine ratio, micro-/macro-albuminuria and eGFR in a group of SCD patients in Cameroon (Geard et al., 2017). Given the high rate of renal dysfunction in SCD patients in Africa with its high genetic diversity, there is need to explore possible novel genetic variants associated with kidney dysfunction. Ideally, whole genome sequencing and other large-scale gene discovery approaches should be use, expanding the targeted genetic discovery to other variants known to be associated with renal dysfunctions. Given the limited number of gene and variant discovery research in SCD patients, a plausible strategy is using renal dysfunctions associated SNPs from non-SCD affected populations.

Several kidney dysfunction genome-wide association studies (GWAS) have been conducted in many non SCD-affected populations (Kottgen et al., 2009, 2010; Chambers et al., 2010; Pattaro et al., 2012). Furthermore, a recent GWAS meta-analysis integrated 15 GWAS studies of 133,413 individuals from multiple ethnicities and uncovered 53 SNPs associated with renal dysfunction, including 26 SNPs found in individuals of African descent (Pattaro et al., 2016). In this study, we investigated the associations of these 26 SNPs in addition to four previously characterised kidney dysfunction-related variants, including *APOL1* (G1 or G2) for *rs60910145, rs73885319* and *rs71785313*, and *HMOX1* for *rs3074372* and *rs743811*, relevant to populations of African ancestry (Pattaro et al., 2016), e.g., SCD patients from Cameroon.

Several studies, including a previous study from our group (Geard et al., 2017), have shown that, in addition to genetic variants, clinical, and biological factors also contribute to glomerular damage (Audard et al., 2017). This highlights the need to employ a multi-factorial approach in investigating factors associated to renal abnormalities in SCD patients. Therefore, in addition to investigating the contribution of the selected 31 SNPs to renal dysfunctions in SCD patients from Cameroon, we also explored the contribution of clinical factors: socio-demographic, anthropometric, clinical and haematological variables, and employed multifactorial regression models for associating the variable to kidney dysfunction parameters. Finally, systems level bioinformatics analyses were performed, employing protein-protein interaction networks to further interrogate the putative associations.

## Materials and Methods

### Ethical Approval

The study was performed following the Declaration of Helsinki. This study was approved by the Faculty of Health Sciences Human Research Ethics Committee of the University of Cape Town, South Africa (HREC REF: 661/2015), and the National Ethics Committee of the Ministry of Public Health, Yaoundé, Republic of Cameroon (No. 193/CNE/SG/10). Patients older than 18 years self-consented into the study and informed consent was given by the parents or guardians for participants younger than 18 years old with a requirement for children older than 7 years to also sign assent forms.

### Study Participants

Cameroonians living with SCD were prospectively recruited at the Yaoundé Central Hospital and Laquintinie Hospital in Douala, between January 2010 and December 2012. Only patients older than 2 years of age who had not received a blood transfusion in the past 6 months were included. None of the patients were receiving *hydroxycarbamide* treatment. Community-based recruitments were also conducted through two SCD patients’ associations who were engaged for collaboration. Additional patients were subsequently recruited during the SCD patient associations’ monthly meetings. No incentive was provided for participation in the study. Socio-demographic and clinical events were collected by means of a structured questionnaire administered to parents/guardians or adult patients. Body mass index (BMI) and blood pressure (BP) were also measured. Patients’ clinical records were extracted from their medical records covering the past 3 years. These clinical records include blood transfusion history, the occurrence of vaso-occlusive crisis (VOC) and hospitalisation rates per year.

### Hematological Phenotypes

Complete blood counts and foetal haemoglobin (HbF) quantifications were conducted during hospital visits. Two methods of HbF detection were employed in this study for patients: initially using the alkali denaturation test in 55% of the cohort and subsequently High-Performance Liquid Chromatography when it became available at the haematological laboratory of the Centre Pasteur of Cameroon (CPC).

### Renal Function Measurements

Urinary albumin quantifications were performed using either the Siemens Clinitek Status test or the Hemocue Albumin 20 system on the first morning urine samples during planned hospital visits, when patients were not experiencing VOC, as previously reported by Geard et al. (2017). The presence of albumin in the urine is defined as normal when the concentration is <30 mg/dl, micro-albuminuria (30–300 mg/dl) or macro-albuminuria >300 mg/dl. The glomerular filtration rate (GFR) is estimated (eGFR) using the Chronic Kidney Disease-Epidemiology Collaboration (CKD-EPI-creatinine) formula. Kidney failure is defined as an eGFR <90 ml/min/1.73 m^2^, renal hyperfiltration as an eGFR > 130 ml/min/1.73 m^2^ for women and >140 ml/min/1.73 m^2^ for men, and normal filtration as an eGFR between 90 ml/min/1.73 m^2^ and 130/140 ml/min/1.73 m^2^ (Haymann et al., 2010).

### Molecular Methods

#### DNA Extraction

DNA was extracted from peripheral blood in EDTA following the manufacturer’s instructions (Puregene Blood Kit) at CPC, and Genotype analyses were performed at the Division of Human Genetics, University of Cape Town, South Africa.

#### Sickle Cell Mutation, Beta-Globin Gene (HBB) Cluster Haplotypes and 3.7 kb Alpha-Globin Gene (HBA1/HBA2) Deletion

Molecular analysis to confirm the presence of the sickle mutation w carried out on 200 ng of DNA by polymerase chain reaction (PCR) to amplify a 770 bp segment of *HBB*, followed by a digestion with DdeI restriction enzyme on the PCR product (Saiki et al., 1985).

Five restriction fragment length polymorphism (RFLP) sites in the *HBB* cluster were amplified using published primers and methods to analyse the HBB haplotype background (Bitoungui et al., 2015).

The 3.7 kb *HBA*_1_/*HBA*_2_ deletion was successfully screened using the expand-long template PCR as previously published (Rumaney et al., 2014).

#### Kidney Dysfunction -Related Targeted Variants

Twenty-six African American specific kidney dysfunction-related gene variants were genotyped in this SCD cohort after being mapped to 53 single nucleotide polymorphisms (SNPs) identified in a GWAS meta-analysis of kidney diseases (Pattaro et al., 2016). Five additional gene variants in *APOL1* and *HMOX1* from the literature were also considered (Genovese et al., 2010; Tzur et al., 2010; Wonkam et al., 2014; Geard et al., 2017; Saraf et al., 2017). Thus, all targeted SNPs in *A1CF-rs10994860, WNT7A-rs6795744, PTPRO-rs7956634, UMOD-rs4293393, LRP2 -rs4667594 ANXA9-rs267734, GCKR-rs1260326, TFDP2-rs3476 85, DAB2-rs11959928, SLC22A2-rs2279463, TMEM60-rs64658 25, SLC6A13-rs10774021, BCAS1-rs17216707, SKIL-rs9682041, UNCX-rs10277115, KBTBD2-rs3750082, CNQ1-rs163160, AP5B 1-rs4014195, NFKB1-rs228611, CACNA1S-rs3850625, SYPL2-rs1 2136063, ETV5-rs10513801, DPEP1-s164748, SIPA1L3-rs1166649 7, NFATC1-rs8091180 and IGFBP5-rs2712184*, and in *APOL1 (G1)-rs60910145, APOL1 (G1)-rs73885319, APOL1 (G2)-rs71 785313, HMOX1-rs3074372, HMOX1-rs743811*, were genotyped with the iPLEX Gold Sequenom Mass Genotyping Array. Thereafter, the validation of the genotyping results was done by Sanger sequencing using BigDye terminator mix in 10% subset of sample (Supplementary Figure S1).

### Statistical and Bioinformatics Analysis

We performed association analyses between kidney dysfunction outcomes (characterised by eGFR and crude-albuminuria scores), socio-demographic and clinical variables, and 31 known kidney dysfunction-related variants among this group of SCD patients. First, to ensure genotypic quality of the data, we ran PLINK 1.9 (Purcell et al., 2007), performing a Hardy-Weinberg Equilibrium (HWE) test with significant level, minor allele frequency (MAF), and missing genotype data thresholds of 0.001, 0.05, and 0.1, respectively. A total of 13 out of 31 SNPs in the dataset did not pass quality control (QC) filters, of which 8 were removed due to missing genotype data and 5 others do not meeting the set minor allele thresholds. With R software, we first performed descriptive statistics to provide a general summary of different parameters to be considered in the analysis. Thereafter, two regression analyses were performed: (1) multi-variable regressions for each kidney dysfunction phenotype with different socio-demographic, anthropometric, haematogical, clinical variables and each genetic variant after adjusting or transforming phenotype values to approximate a symmetric (normal) distribution based on their Fisher-Pearson skewness coefficient scores; (2) logistic regressions for each kidney dysfunction phenotype and all variables and genetic variants under consideration after mapping phenotypes to 0 (controls) or 1 (cases: micro- and macro-albuminuria for crude-albuminuria, then renal failure and hyperfiltration for eGFR). Finally, we performed functional and protein–protein interaction network enrichment analyses, using Gene Ontology (GO) process (The Gene Ontology Consortium, 2019), the protein GO Annotation (GOA) mapping (Mi et al., 2019) and the Kyoto Encyclopeadia of Genes and Genomes (KEGG) pathway (Kanehisa et al., 2019) datasets, to identify potential enriched biological processes and pathways in which identified candidate genes are involved. A significance level of 0.05 was considered after adjusting *p*-values (P) for Bonferroni multiple corrections and gene functional annotations were retrieved from the Ensembl database (Yates et al., 2020).

## Results

### Description of Study Participants

A total of 413 SCD steady state Cameroonian patients were included in the study. The participants’ characteristics are described in Table 1. There are roughly equal numbers of males and females (M/F = 210/203), with a median age of 15.5 years. The median number of VOC per year was 2 (range: 0–80). 41% (*n* = 168) of patients had (3 VOC per year and 28.1% (n (=114) had 2 hospitalizations hospitalisations per year. All participants were homozygous HbSS, Benin being the most prevalent (-globin-like gene cluster haplotype (55%; *n* = 195). It is worth noting that, in this study, we used the modified annotation protocol suggested by Crawford et al. (2002) and Hanchard et al. (2007) for determining β-globin gene cluster haplotypes Benin, Cameroon, Bantu, Senegal and Arab-Indian. Haplotypes that are not conform to these are considered to be “Atypical.” We refer to “Other haplotypes,” all underrepresented haplotypes in the cohort, covering Bantu, Senegal, and Arab-Indian. 32 and 11% co-inherited a single or double 3.7 kb *HBA*_1_/*HBA*_2_ deletion, respectively.

**TABLE 1:** Description of the Cameroonian SCD cohort.

Variables	Median (95% CI) or frequency (%)	Min–max	*P*-value	Observation (*n*)
Age (years)	15.5 (15–16.5)	2–58	0.1123	413
Gender	M/F: 210/203			413
**Clinical Events**				
VOC (*n*/year)	2 (2–3)	0–80	0.4949	412
Hospitalisation (*n*/year)	1 (1–1)	0–40	0.2676	405
Transfusion	Y/N: 76.8/23.2			410
**Anthropometric**				
Body mass index (kg/m^2^)	17.6 (17.2–17.9)	10.6–32	0.1155	413
Systolic blood pressure (mmHg)	103 (101.5–104)	72–156	0.8637	409
Diastolic blood pressure (mmHg)	58 (57–59)	37–93	0.0162	409
**Haematological**				
Hb (g/dl)	7.55 (7.4–7.7)	3.5–13.1	0.4905	406
MCV (fl)	91 (90–92)	61–125	0.9905	406
Platelets (10^9^/l)	368.5 (355–382)	29–1,078	0.8483	406
Leucocytes (10^9^/l)	12.65 (12.2–13.2)	4–49.8	0.0471	406
Lymphocytes (10^9^/l)	5.2 (4.95–5.4)	1.4–22.1	0.0219	406
Monocytes (10^9^/l)	1.5 (1.45—-1.6)	0.1–8.1	0.0009	406
Granulocytes (10^9^/l)	4.5 (4.25–4.75)	0.2–24.3	0.0976	406
HbA2 (%)	3.15 (2.9–3.3)	0–18.2	0.0008	412
HbF (%)	9.95 (9.3–10.65)	0–37.2	0.2026	412
**Alpha-thalassemia Genotypes (%)**			<0.0001	339
αα/αα	57			194/339*
αα/α3.7	32			109/339*
α3.7/α3.7	11			36/339*
**HBB Haplotype (%)**			<0.0001	352
Ben/Ben	55			195/352*
Ben/Cam	26			92/352*
Ben/Atypical	7			25/352*
Cam/Cam	7			23/352*
Cam/Atypical	2			7/352*
Atypical	1			3/352*
Other haplotypes	2			7/352*
**Renal functions**				
Serum creatinine (mg/l)	6.8 (6.5–7)	2–13.8	0.3958	404
Crude-albuminuria (mg/dl)	51.5 (47–55.5)	3–1,180	<0.0001	407
*Normal (%)*	37			149/407
*Micro-albuminuria (mg/dl) (%)*	61			248/407
*Proteinuria (mg/dl) (%)*	2			10/407
eGFR (ml/min/1.73 m^2^)	155.4 (151.6–159.2)	58.9–290.7	0.5715	404
*Normal (%)*	23			93/404
*Glomerular hyperfiltration (%)*	71			287/404
*Kidney failure (%)*	6			24/404

*Number of individuals, not alleles. VOC, vaso-occlusive crises; Hb, haemoglobin; MCV, mean corpuscular volume; HbA_2_, Haemoglobin A_2_; eGFR, estimated glomerular filtration rate. In all cases, Wilcoxon tests were performed to check whether the parameter falls within the confidence interval (CI) for p-value greater than 0.05, except for alpha-thalassemia genotypes and the HBB Haplotype, for which adjusted (χ2 p-values were computed to check whether these proportions follow a uniform distribution for p-value *greater than 0.05. In the second column, median values are followed by CI, whereas proportions are not.*

### Clinical and Socio-Demographic Factors Associated With Renal Dysfunctions

**FIGURE 1:**
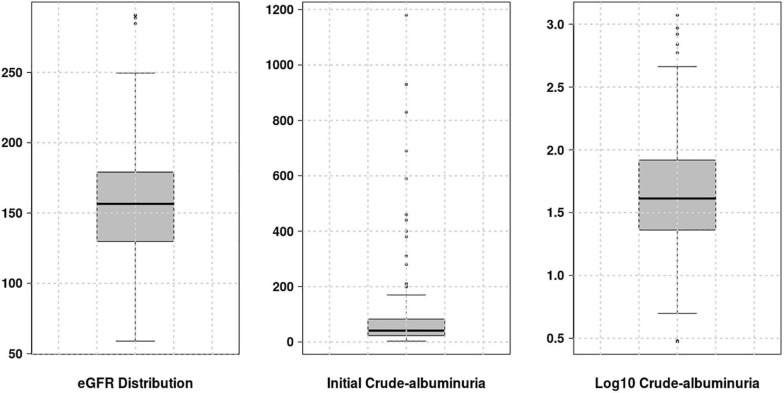
Distribution of different phenotype values. For eGFR, no transformation was required, however, for crude-albuminuria, the initial distribution is highly skewed and log10 transformation was applied to approximate a normal distribution.

For the genotype dataset and two renal phenotypes under consideration, a prior QC and value adjustment processes were performed, respectively. Only 18 out the 31 genetic variants (highlighted in Table 2) were considered for further analyses: eight variants were removed due to missing genotype data (genotype call rate 0.9: for cut-off of 0.1) and 5 had minor allele frequency of less than 5%. For each phenotype to approximate a symmetric or normal distribution, we computed the Fisher-Pearson skewness coefficients to select the type of transformation required. For eGFR, no value adjustment was required as its skewness coefficient was 0.27581, comprised in the range of -0.5 and 0.5 fitting a symmetric distribution. For crude-albuminuria, the skewness coefficient is 6.45456 ≥ 1, in which case, the log10 transformation was applied to ensure that crude-albuminuria dataset approximate a symmetric distribution (Figure 1).

**TABLE 2:** Allele frequencies of kidney dysfunction-related gene variants.

Gene	dbSNP ID	SNP position	Allele change	MAF	Proven disease associations (Ensembl)
***UNCX***	rs10277115	7:1245559	A > T	0.16	Renal function related trait
***APOL1 (G1)***	rs73885319	22:36265860	T > G	0.13	Renal function related trait
***APOL1 (G2)***	rs71785313	22:36266000	TTATAA > Deletion	0.082	Renal function related trait
***A1CF***	rs10994860	10:50885664	C > T	0.24	Glomerular filtration rate
***DAB2***	rs11959928	5:39397030	T > A	0.32	Chronic kidney disease
***SYPL2***	rs12136063	1:109471548	G > A	0.29	Glomerular filtration rate
***GCKR***	rs1260326	2:27508073	C > T	0.057	-
***KCNQ1***	rs163160	11:2768725	A > G	0.057	Glomerular filtration rate
***SLC22A2***	rs2279463	6:160247357	A > G	0.23	Chronic kidney disease
***NFKB1***	rs228611	4:102640552	G > A	0.228	Glomerular filtration rate
***IGFBP5***	rs2712184	–	C > A	0.46	–
***TFDP2***	rs347685	3:142088295	A > C	0.248	Chronic kidney disease
***UMOD***	rs4293393	16:20353266	A > G	0.227	Chronic kidney disease
***TMEM60***	rs6465825	7:77787122	C > T	0.45	Chronic kidney disease
***WNT7A***	rs6795744	3:13865353	G > A	0.191	Glomerular filtration rate
***PTPRO***	rs7956634	12:15168260	C > T	0.45	Glomerular filtration rate
***NFATC1***	rs8091180	18:79404243	G > A	0.068	Glomerular filtration rate
***SKIL***	rs9682041	3:170374114	T > C	0.274	Glomerular filtration rate

After the transformation, we performed linear regression models and factors associated with crude albuminuria and eGFR in Cameroonian SCD patients are presented in Table 3. Age and gender as well as creatinine were not considered in the eGFR-based regression models to prevent biases in outputs as these two factors are confounders, mixing up with eGFR as an outcome (because age, gender and creatinine contribute to the eGFR calculation).

**TABLE 3:** Blood pressure, clinical, and haematological variables, and genetic variants associated eGFR and Crude albuminuria.

*eGFR* (ml/min/1.73 m^2^)	Effect size (SE)	Mean variation explained (%)	*P*-values
SBP	−15.01547 (7.51850)	40.55	0.04674
VOC	0.79123 (0.35834)	21.73	0.02802
Hb	3.11861 (1.40541)	16.44	0.02725
MCV (fl)	0.68573 (0.15854)	1.90	2.09130e–05
HbF	1.62340 (0.32960)	1.13	1.40989e–06
Platelet	0.05052 (0.01497)	0.28	8.35290e–04
Granulocytes	2.06830 (0.76779)	0.12	7.47090e–03
Lymphocytes	2.12071 (0.97584)	0.08	0.03057
rs12136063*	0.57907 (0.28487)	11.04	0.04208
rs10994860*	−0.69852 (0.30075)	0.18	0.02020
rs73885319*	0.890305 (0.44638)	0.97	0.04610
Crude albuminuria (mg/dl)			
BMI	0.01872 (0.00596)	45.34	1.87777e–03
MCV (fl)	0.01273 (0.00121)	1.22	1.17630e–07
Transfusion	0.12406 (0.05602)	0.23	0.02759
Hb*	−0.28340 (0.11040)	0.19	0.0103
rs6795744*	−0.59520 (0.28580)	3.70	0.03730
rs6465825*	−0.46910 (0.20690)	0.65	0.02340
rs71785313	−0.12686 (0.06086)	0.10	0.03803

Effect provides an indication on the influence of a given factor on the phenotype under consideration and mean variation explained the average contribution of a specific factor to the phenotype. SE stands for standard error and * indicates that the association was inferred from logistic model.

#### eGFR

**FIGURE 2:**
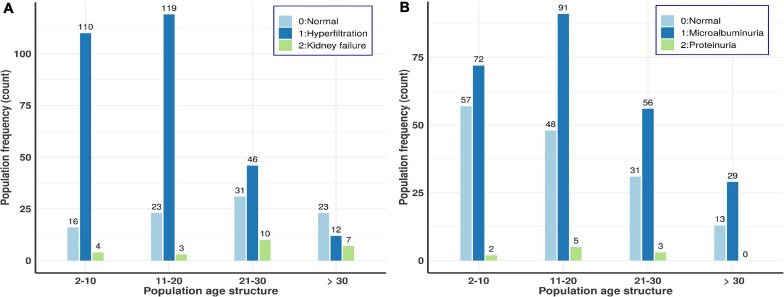
Age-based population distributions for the two kidney dysfunction indicators, i.e., crude-albuminuria and eGFR scores. **(A)** Age-based population distribution using eGFR scores. **(B)** Age-based population distribution using microalbuminuria scores.

the level of serum creatinine used to estimate GFR had a median of 6.8 mg/l and the eGFR median was 155.4 ml/min/1.73 m^2^ (Table 1). Up to 71% of the patients had glomerular hyperfiltration and 5.9% renal failure (see overall age-based population distributions in Figure 2A). Figure 2A suggests that the prevalence of glomerular hyperfiltration is high amongst patients under 21 years old and kidney failure is relatively high up to 30 years, decreasing after 30 years. This agrees with another Canadian study which highlighted that children with SCD between 4 and 11 years have a significantly higher mean eGFR (Mammen et al., 2017). The eGFR was significantly increased in male patients (*P* = 4.65297e–10). Isolated hyperfiltration was present in 25.8% (*n* = 105) of patients, while 41.5% (*n* = 169) and 2.2% (*n* = 9) were experiencing glomerular hyperfiltration with micro- and macro-albuminuria, respectively. Haemoglobin, HbF, MCV, platelet, lymphocytes, and granulocytes presented highly significant *p*-values and positive correlation with eGFR, explaining some proportion of eGFR variations (Table 3). This suggested that there was a reduced protection of kidney from haemoglobin mediated toxicity. Though high HbF level as well as lymphocytes and granulocytes have beneficial clinical effect on SCD patients and results indicated that an increased level of these variables beyond the steady state likely increase the risk of clinical complications. Finally, eGFR is also correlated to systolic BP (*P* = 0.04674) and VOC frequencies (*P* = 0.02802), explaining 40.55 and 21.73% of the eGFR variation, respectively (Table 3), with stable systolic BP being highly protective against glomerular hyperfiltration.

##### Crude-Albuminuria

The median of crude-albuminuria was 51.5 mg/dl ranging from 3 to 1,180 mg/dl. The prevalence of micro and macro-albuminuria was 61 and 2%, respectively. All age ranges have nearly equal proportions of micro-albuminuria, while proteinuria is shown to increase with age (Figure 2B). Figure 2B shows similar profile as for eGFR in Figure 2A and indicates that the prevalence of microalbuminurea is high amongst patients under 21 years old, with some proteinurea cases that vanish after 30 years of ages. This also agrees with another cohort study in Ghana (Anto et al., 2019), which indicated that the prevalence of renal complications, such as proteinuria, is high in young patients aged between 5 and 12 years. BMI, MCV, haemoglobin and transfusion are significantly associated with crude albuminuria with the highest phenotypic variation explained by BMI (45.34%) and Haemoglobin with reduced risk of micro-albuminuria, though with small effect size (Table 3).

### Associations of eGFR and Crude-Albuminuria With Kidney Disfunction-Related Gene Variants

**FIGURE 3:**
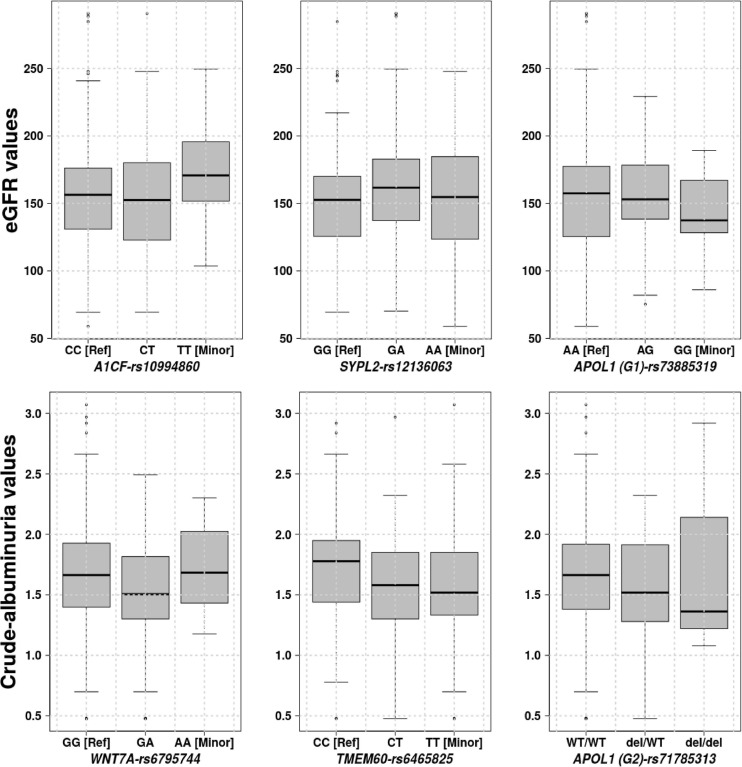
Phenotype values, eGFR and crude-albuminuria values based on the significant gene variants distributed over homozygous dominant, recessive, and heterozygous genotypes.

Three genetic variants: *Synaptophysin-like protein 2* (*SYPL2-rs12136063*), *APOBEC1 complementation factor* (*A1CF-rs10994860*) and *Apolipoprotein L1* (*APOL1 (G1)-rs73885319*) are significantly associated with eGFR with respective *P*-values of 0.04208, 0.02020, and 0.04610. Figure 3 shows the eGFR values for three gene variants distributed in homozygous dominant, recessive, and heterozygous genotypes. These distribution values suggested that the two allele changes (homozygous recessive) of *A1CF-rs10994860* is protective against renal dysfunction (Table 3). On the other hand, one allele change or heterozygous genotype showed a protective effect against prevalent hyperfiltration considering the negative correlation with eGFR (conferring about 2 times more protection as compared to a patient with no copy). *SYPL2-rs12136063* confers increased risk of progressing to the renal dysfunction and *APOL1 (G1)-rs73885319* to prevalent hyperfiltration and ultimately to the renal dysfunction (approximately 2 times more likely to progress to renal dysfunction).

Three genetic variants were also identified to be significantly associated with crude-albuminuria: *Protein Wnt-7a (WNT7A-rs6795744)*, *Transmembrane protein 60 (TMEM60-rs6465825)*, *APOL1 (G2)-rs71785313* with *P*-values of 0.03730, 0.02340, and 0.03803, respectively. With knowledge of the crude-albuminuria distribution as shown in Figure 3, a change in allele or WT deletion in the case of *APOL1* provides a protective effect against prevalent micro-albuminuria (Table 3). These results indicate that patients with *WNT7A-rs6795744* and *TMEM60-rs6465825* changes are approximately 2 times less likely to progress to micro-albuminuria state, and a single and double WT *APOL1* (G2) deletions decrease crude-albuminuria value by 0.12686 and 0.24732 ml/min/1.73/m^2^, respectively. Though this phenotype level change is negligible due to the effect size, it can be essential for crude-albuminuria extreme values (e.g., values on the border line of state changes).

### Distribution of HbF Levels vs. β-Globin Gene Cluster Haplotypes and α-Globin Gene Deletions

**FIGURE 4:**
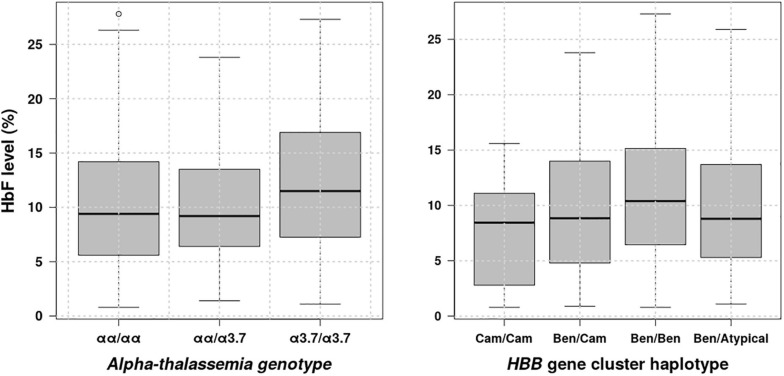
Distribution of HbF levels vs. β-globin gene cluster haplotypes and α-globin gene deletions.

HbF is a major SCD modifier, which is known to modulate the SCD phenotype (Coleman and Inusa, 2007), to ameliorates pathophysiological and clinical manifestations of the sickling process (Adekile, 2020). There is accumulating evidence indicating that this major disease modifier is influenced by β-globin gene cluster haplotypes (Lakkakula et al., 2017; Piel et al., 2017; Adekile, 2020) and (α-globin gene deletions (Piel et al., 2017). Thus, we have looked at distribution of fetal haemoglobin levels vs. representative β-globin gene cluster haplotypes and α-globin gene deletions and results are shown in Figure 4. These results indicate that HbF levels vary in the population based on β-globin gene cluster haplotypes and α-globin gene deletions. Individuals with Benin haplotyte and double 3.7 kb α-globin gene deletions having significantly higher HbF levels with *P* = 0.0813e–2 and 0.0203, respectively, accepting the alternative hypothesis that the HbF level median is greater than 9.3%, the lower bound of the HbF level confidence interval (Table 1), which is slightly higher than the minimal level (8.6%) indicated to improve SCD patient survival (Coleman and Inusa, 2007). Furthermore, we checked whether there is any association between exposure to Benin haplotype or double 3.7 kb α-globin gene deletions and outcomes, namely renal dysfunction phenotypes: eGFR and albuminurea. Results obtained have revealed an association between double 3.7 kb α-globin gene deletions and eGRF showing a protective effect against prevalent hyperfiltration/kidney failure with odds ratio = 0.36320 (95%CI: 0.16695–0.80532, *P* = 0.01007) under the null hypothesis that odds ratio is equal to 1.

### Selecting Optimal Phenotype Proxy for Kidney Dysfunction Prediction

**FIGURE 5:**
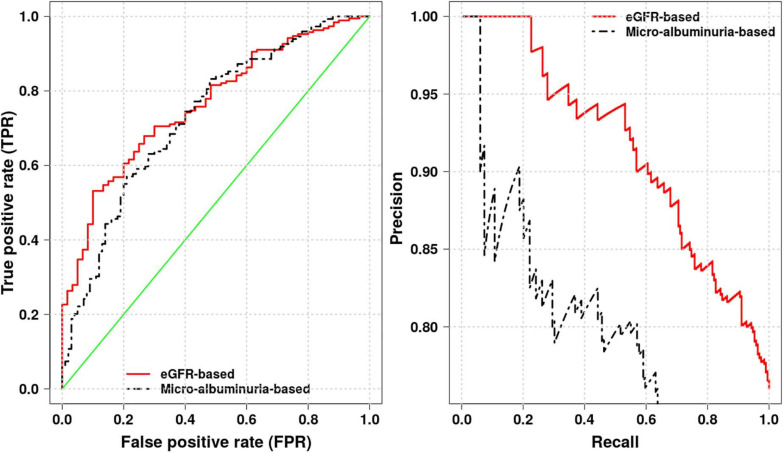
Receiver operating characteristic (ROC) and Precision-Recall curves for eGFR- and crude-albuminuria-based logistic models.

Using the eGFR- and crude-albuminuria-based logistic regression model, we compute areas under the receiver operating characteristic (ROC) and Precision-Recall (PR) curves, as well as accuracy to identify the best phenotype proxy for predicting kidney dysfunction in Cameroonian SCD patients. Different areas are shown in Figure 5, with eGFR-based model achieving the area under ROC of 0.76 and an accuracy score of 0.78 vs. the area under ROC of 0.73 with an accuracy score of 0.70 for crude-albuminuria-based model. This suggests that it is more effective to use eGFR phenotype as a proxy for predicting kidney dysfunction in Cameroonian SCD population. This is also in agreement with the Akaike’s Information Criterion (AIC) scores produced by the two models, 284.33 for eGFR-based model vs. 361.32 for crude-albuminuria-based model, indicating that eGFR-based model fits data better than crude-albuminuria phenotype proxy. This suggests that a simple classification learning algorithm can be designed, taking as inputs, patient age, gender, and level of serum creatinine and predicting kidney dysfunction in patients.

### Identifying Essential Genes and Functional Enrichment Analyses

**FIGURE 6:**
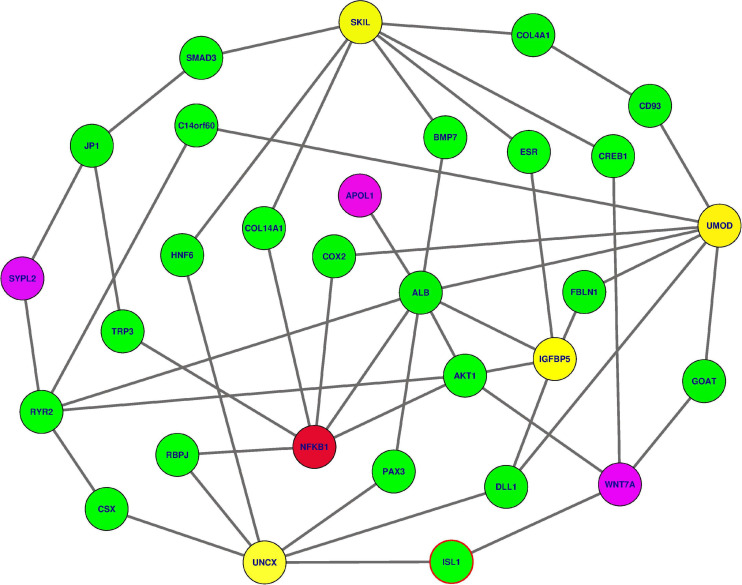
The subnetwork extracted from the human PPI network revealing how predicted gene variants interact together to influence the kidney dysfunction (refer to Table 4 for gene descriptions). In yellow and magenda are gene variants previously shown to be associated with kidney-dysfunction, three in magenda color have been confirmed. In green color, are intermediate nodes used by kidney-dysfunction gene variants to reach the one in red color (*NFKB1*) indicated to be essential in the PPI network.

We analysed how interactive genes from knowledge-based Protein-Protein Interaction (PPI) interacted with the 18 genes used in different analyses, focusing specifically on six genes identified to be associated with kidney dysfunction phenotypes. Mapping these 18 genes to a comprehensive human Protein-Protein Interaction (PPI) network (Wu et al., 2009; Mazandu et al., 2018), we identify sub-networks containing these gene variants: *APOL1, SYPL2, WNT7A*, *IGFBP5*, *UNCX*, *NFKB1*, *UMOD*, and *SKIL*. Three gene variants within this sub-network, namely APOL1 (G1)-rs73885319, as well as APOL1 (G2)-rs71785313, WNT7A-rs6795744, and SYPL2-rs12136063, have been identified to influence variation in renal dysfunction phenotypes in SCD patients. These variant genes are connected to *NFKB1*, identified to be essential or a hub based on the network centrality measures within the sub-network via some specific intermediate genes (Figure 6 and Table 4), following a small world property of human PPI network (Mazandu et al., 2020). This *NFKB1* gene might indirectly influence extreme phenotype levels. Moreover, these gene variants are enriched with the *cartilage condensation process* (*P* = 0.02976) in which *WNT7A*, revealed to be likely implicated in SCD patient renal dysfunction, is involved. This process is possibly involved in the development of VOC complications, resulting in proteinuria and glomerular hyperfiltration, and ultimately in kidney damage.

**TABLE 4:** The description of the different genes displayed in Figure 6 extracted from the UniProt database (https://www.uniprot.org/).

Gene	UniProt description
*COL4A1*	Collagen alpha-1 (IV) chain (Cleaved into: Arresten)
*ISL1*	Insulin gene enhancer protein ISL-1 (Islet-1)
*CD93*	Complement component C1q receptor (C1q/MBL/SPA receptor) (C1qR) (C1qR(p)) (C1qRp) (CDw93) (Complement component 1 q subcomponent receptor 1) (Matrix-remodeling-associated protein 4) (CD antigen CD93)
SMAD3	Mothers against decapentaplegic homolog 3 (MAD homolog 3) (Mad3) (Mothers against DPP homolog 3) (hMAD-3) (JV15-2) (SMAD family member 3) (SMAD 3) (Smad3) (hSMAD3)
*AKT1*	RAC-alpha serine/threonine-protein kinase (EC 2.7.11.1) (Protein kinase B) (PKB) (Protein kinase B alpha) (PKB alpha) (Proto-oncogene c-Akt) (RAC-PK-alpha)
*JP1*	Junctophilin-1 (JP-1) (Junctophilin type 1)
*FBLN1*	Fibulin-1 (FIBL-1)
*COL14A1*	Collagen alpha-1 (XIV) chain (Undulin)
*COX2*	Prostaglandin G/H synthase 2 (EC 1.14.99.1) (Cyclooxygenase-2) (COX-2) (PHS II) (Prostaglandin H2 synthase 2) (PGH synthase 2) (PGHS-2) (Prostaglandin-endoperoxide synthase 2)
*RBPJ*	Recombining binding protein suppressor of hairless (CBF-1) (J kappa-recombination signal-binding protein) (RBP-J kappa) (RBP-J) (RBP-JK) (Renal carcinoma antigen NY-REN-30)
*RYR2*	Ryanodine receptor 2 (RYR-2) (RyR2) (hRYR-2) (Cardiac muscle ryanodine receptor) (Cardiac muscle ryanodine receptor-calcium release channel) (Type 2 ryanodine receptor)
*UNCX*	Homeobox protein unc-4 homolog (Homeobox protein Uncx4.1)
*C14orf60*	Neurexin-3 (Neurexin III-alpha) (Neurexin-3-alpha)
*NFKB1*	Nuclear factor NF-kappa-B p105 subunit (DNA-binding factor KBF1) (EBP-1) (Nuclear factor of kappa light polypeptide gene enhancer in B-cells 1) (Cleaved into: Nuclear factor NF-kappa-B p50 subunit)
*HNF6*	Hepatocyte nuclear factor 6 (HNF-6) (One cut domain family member 1) (One cut homeobox 1)
*ESR*	Estrogen receptor (ER) (ER-alpha) (Estradiol receptor) (Nuclear receptor subfamily 3 group A member 1)
*UMOD*	Uromodulin (Tamm-Horsfall urinary glycoprotein) (THP) (Cleaved into: Uromodulin, secreted form)
*CREB1*	Cyclic AMP-responsive element-binding protein 1 (CREB-1) (cAMP-responsive element-binding protein 1)
*DLL1*	Delta-like protein 1 (Drosophila Delta homolog 1) (Delta1) (H-Delta-1)
*SKIL*	Ski-like protein (Ski-related oncogene) (Ski-related protein)
*PAX3*	Paired box protein Pax-3 (HuP2)
*IGFBP5*	Insulin-like growth factor-binding protein 5 (IBP-5) (IGF-binding protein 5) (IGFBP-5)
*BMP7*	Bone morphogenetic protein 7 (BMP-7) (Osteogenic protein 1) (OP-1) (Eptotermin alfa)
*SYPL2*	Synaptophysin-like protein 2
*ALB*	Fas-binding factor 1 (FBF-1) (Protein albatross)
*TRP3*	Short transient receptor potential channel 3 (TrpC3) (Transient receptor protein 3) (TRP-3) (hTrp-3) (hTrp3)
*APOL1*	Apolipoprotein L1 (Apolipoprotein L) (Apo-L) (ApoL) (Apolipoprotein L-I) (ApoL-I)
*WNT7A*	Protein Wnt-7a
*GOAT*	Ghrelin O-acyltransferase (EC 2.3.1.-) (Membrane-bound O-acyltransferase domain-containing protein 4) (O-acyltransferase domain-containing protein 4)
*CSX*	Homeobox protein Nkx-2.5 (Cardiac-specific homeobox) (Homeobox protein CSX) (Homeobox protein NK-2 homolog E)

## Discussion

This is the first study to investigate the relevance of kidney dysfunction-related variants identified through a GWAS meta-analysis as well as functional enrichment and protein-protein interaction network analyses in SCD patients. The results highlighted the high prevalence of micro-albuminuria as presented in a previous study in Cameroon by Geard in 2017 (Geard et al., 2017). This prevalence is much higher than the values of 18.5 and 27% observed in paediatric cohorts from several sub-Saharan African countries (Ranque et al., 2014; Aloni et al., 2017), the 13.2% in the multicentric study of children with SCD in the United States (Schaefer et al., 2016) and the 44% in adults from Nigerian and the United States (Bolarinwa et al., 2012; Drawz et al., 2016). These differences likely reflect the lack of appropriate care of SCD or the manifestation of the most severe SCD phenotype in Cameroon. The low proportion of macro-albuminuria found in this study is distinct from 15.1% reported in a cohort of SCD patients in the United Kingdom (UK) (Brewin et al., 2017). This could be due to the difference in age structure between the two cohorts. This study replicated a positive association of crude-albuminuria with increasing age as presented by a multi-center African study (Ranque et al., 2014) and a Nigeria-based study (Brewin et al., 2017). Some haematological variables, such as MCV and Hb level (Table 3), influence crude albuminuria among SCD patients from Cameroon, Hb level observed to be protective against micro- albuminuria. This is in accordance with studies in Jamaica and the United States (Aban et al., 2017; Niss et al., 2020) which revealed that lower concentration of Hb is associated with development of micro-albuminuria, leading to relative renal ischemia, ischemia-reperfusion injury, and increased medullary sickling (Aban et al., 2017). BMI provides the highest mean variation explained for crude-albuminuria and may be a major anthropometric factor leading to renal dysfunction. This is likely related to the nutrition of SCD patients who struggle to maintain an adequate quality of life. We have not observed any significant association of crude-albuminuria with WBC counts or BP as previously observed in a Jamaican cohort (Asnani et al., 2016).

The prevalence of glomerular hyperfiltration was similar to the 76% found among SCD children in the United States (Aygun et al., 2011), but higher than that previously reported in France (51%) (2010) (Haymann et al., 2010) and DRC (2017) (Aloni et al., 2017). It was lower than the 98% presented in the United Kingdom (Drawz et al., 2016). These differences may be explained by the variability in the median ages of participants in the other studies; they may also be due to the method used to calculate eGFR, as the Schwartz formula tends to underestimate GFR in children compared to the CKD-EPI used in the present study. Unlike the studies of Asnani in Jamaica (Aban et al., 2017) and Vazquez in the United States (Aygun et al., 2011), no significant associations between eGFR and crude albuminuria were identified in this study. However, 41 and 2% of patients had glomerular hyperfiltration associated with micro-albuminuria and proteinuria, respectively. These results are different from the values highlighted by Haymann et al. (2010) and from the 22% of patients who had both glomerular hyperfiltration and micro-albuminuria in DRC (Aloni et al., 2017). The eGFR was highly associated with haemoglobin, HbF and SBP, as previously observed in patients from the United Kingdom (Wu et al., 2009). The strong association between SBP and eGFR is also consistent with data reported in the study by Niss et al. (2020) in Jamaica confirming the highly protective role of stable SBP against glomerular hyperfiltration (Table 3). VOC frequencies are also highly correlated to eGFR, explaining the morbidity due to renal dysfunction among SCD patients in Cameroon. Associations between eGFR with Hb level and WBC (granulocytes and lymphocytes) were previously highlighted by Aban et al. (2017), revealing that a low Hb level and increased WBC are associated with renal failure. The correlations between eGFR with age, BMI and creatinine were observed in patients from the United Kingdom (Drawz et al., 2016), Jamaica (Eke et al., 2012), Nigeria (Ajite et al., 2019), and the United States (Becker et al., 2014). However, age and creatinine were not confirmed by this study as they were confounding factors, mixing up with eGFR. This suggests that previous observations were biased, and results obtained by these studies may be flawed. Our analysis agreed with publications within the Taiwanese (Chang et al., 2018) and Caucasian (Brown et al., 2012) which found no association between BMI and renal dysfunctions.

Results highlighted that only three replicated genetic variants are associated to renal dysfunction: *A1CF-rs10994860* with reduced risk of renal dysfunction for two allele changes and a protective effect against prevalent glomerular hyperfiltration; It confers about 2 times more protection to SCD individuals with one allele change or heterozygous genotype as compared to a patient with no copy, and explaining only 0.18% of status change to the glomerular hyperfiltration or renal dysfunction. However, *SYPL2-rs12136063* as well as *APOL1 (G1)-rs73885319* conferred increased risk of progressing to the renal dysfunction or to prevalent glomerular hyperfiltration, explaining 11.04 and 0.97% of status changes, respectively. Like eGFR, three gene variants are also identified for crude-albuminuria: *WNT7A-rs6795744, TMEM60-rs6465825*, and *APOL1 (G2)-rs71785313* providing a protective effect against prevalent micro-albuminuria (Table 3), explaining less than 4% of status changes. These relative low contributions to status changes suggests that the targeted SNPs may not be relevant to the ancestral African populations, or it is possible SCD specific kidney dysfunctions associated variants are still to be found. Indeed, there is bias in polygenic risk scores (PRSs) regarding usability, and transferability for complex trait, as most PRSs do not account for multiple alleles that are either limited or of high frequency among Africans, due highest genomic variations (Gurdasani et al., 2019). A genome-wide association study (GWAS) on genetic susceptibility to type 2 diabetes (T2D) identified a previously unreported African-specific significant locus, while showing transferability of 32 established T2D loci (Adeyemo et al., 2019). Alternatively, kidney dysfunction in SCD may be mostly driven by the pathophysiology of the disease itself rather than genetic factors. For eGFR, SBP explained this status changes by nearly 40.55%, followed by the number of VOC (21.73%), Hb (16.55%), and the upper out range level of HbF would explain the status changes for nearly 1.13%. The HbF level distribution results indicate that HbF levels vary with β-globin gene cluster haplotypes, as well as with α-globin gene deletions in agreement with the current knowledge. The HbF level in patients with Benin haplotyte and double 3.7 kb α-globin gene deletions is significantly higher as compared to the HbF level cutoff observed to improve the patient survival. Thus, these two genetic events may confer a relatively favorable clinical manifestation (Steinberg, 2009; Bitoungui et al., 2015) and patients with double 3.7 kb α-globin gene deletions are about 3 times less likely to progress to the glomerular hyperfiltration or kidney failure state as compared to a patient with no or one deletion (odds ratio = 0.36320). For crude-albuminuria, the status change is mainly explained by BMI with approximately 45.34% of variations, followed by MCV with 1.22%, transfusion (0.23%), and Hb (0.19%).

## Limitations

The first limitation of the present study is the cross-sectional design. A longitudinal study would give more precise data on kidney dysfunction in SCD in Cameroon. Another limitation is the use of the CKD-EPI-creatinine equation to estimate GFR. Recent reports indicate that the CKD-EPI original formula overestimates GFR values. The reliability of the CKD-EPI equation was recently adjusted by inclusion of a molecular weight protein, Cystatin C (CysC), which is eliminated exclusively by glomerular filtration (Yee et al., 2017). However, the protein was not quantified for this cohort. The imbalanced distribution of individuals without kidney dysfunction in this group of SCD patients likely affects the performance of the different regression models (Jedrzejowicz et al., 2018), tending to be biased toward the normal ranges (KrishnaVeni and Sobha, 2011) and potentially failing to identify possible signals.

## Conclusion

This study has replicated *APOL1* gene variants: *(G1)-rs73885319* and *(G2)-rs71785313*, shown to be strongly associated with renal dysfunction in SCD patients, as well as *A1CF-rs10994860*, *SYPL2-rs12136063*, *WNT7A-rs6795744*, and *TMEM60-rs6465825* in Cameroonian SCD patients. Though the protein-protein interaction network and enrichment analyses have revealed the sub-network, which may influence extreme phenotype levels, enriched with the *cartilage condensation process*, which likely contributes to the development of VOC complications and possibly to the renal dysfunction, these gene variants only explain a small proportion of status changes. The results also suggest that haematological indices, clinical events, anthropometric and socio-demographic variables, are major contributors to the pathophysiology of kidney dysfunction in SCD. This elicits the need for further research to investigate new genetic biomarkers which account for kidney dysfunction risk factors in SCD patients in the African setting.

## Data Availability Statement

The data supporting the findings of this study are available from the corresponding author upon reasonable request.

## Ethics Statement

The studies involving human participants were reviewed and the study was performed following the Declaration of Helsinki. This study was approved by the Faculty of Health Sciences Human Research Ethics Committee of the University of Cape Town, South Africa (HREC REF: 661/2015), and the National Ethics Committee of the Ministry of Public Health, Yaoundé, Republic of Cameroon (No. 193/CNE/SG/10). Patients older than 18 years self-consented into the study and informed consent was given by the parents or guardians for participants younger than 18 years old with a requirement for children older than 7 years to also sign assent forms. Written informed consent to participate in this study was provided by the participants’ legal guardian/next of kin.

## Author Contributions

AW, VN-B, KM, TM, and GA conceived and designed the experiments. VN-B, SB, KM, and TM performed the experiments. VN-B, SB, RE, FN-S, TM, KM, and AW needed patient recruitment, samples, and clinical data collection. GM, VN-B, VN, KM, and AW processed and analysed the data. AW, GA, GM, and VN contributed to reagents, materials, and analysis tools. VN-B, KM, GM, and AW wrote the manuscript. GM, VN-B, SB, KM, TM, RE, VN, FN-S, GA, and AW revised and approved the manuscript. All the authors approved the manuscript and agreed to be accountable for all aspects of the presented work.

## Conflict of Interest

The authors declare that the research was conducted in the absence of any commercial or financial relationships that could be construed as a potential conflict of interest.

## Abbreviations

SCD: sickle cell disease
eGFR: estimated glomerular filtration rate
VOC: vaso-occlusive crisis
GWAS: genome-wide association studies
BMI: Body mass index
BP: blood pressure
HbF: foetal haemoglobin
HBB: beta-globin gene
HBA: alpha-globin gene
PCR: polymerase chain reaction
RFLP: Restriction fragment length polymorphism
WBC: white blood cell
Hb: haemoglobin
MCV: mean corpuscular volume
CKD-EPI: Chronic Kidney Disease-Epidemiology.
