# Supplementary material for: Investigations of Kidney Dysfunction-Related Gene Variants in Sickle Cell Disease Patients in Cameroon (Sub-Saharan Africa)
Source: Front Genet. 2021 Mar 15;12:595702. doi: 10.3389/fgene.2021.595702 (PMC8005585; doi:10.3389/fgene.2021.595702)
Supplement: Supplementary file 1 [file Data_Sheet_1.docx]

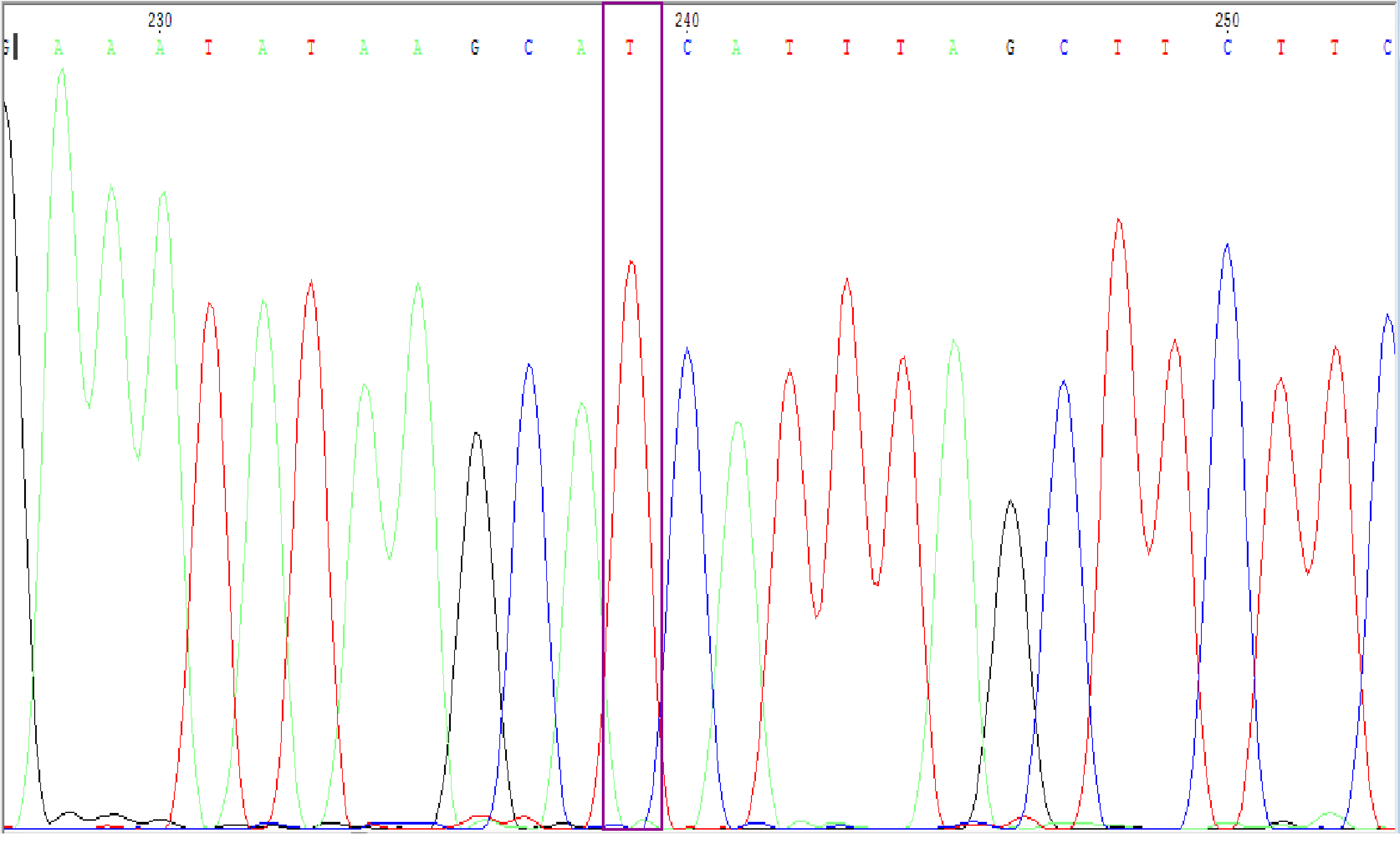


**Fig S1**: Electrogram produced using ABI Prism 3130xl Genetic Analyser (Applied Biosystems). Analysis was performed using the software program Chromas (Version 2.6.6). This is the genotype for rs7956634 C to T.


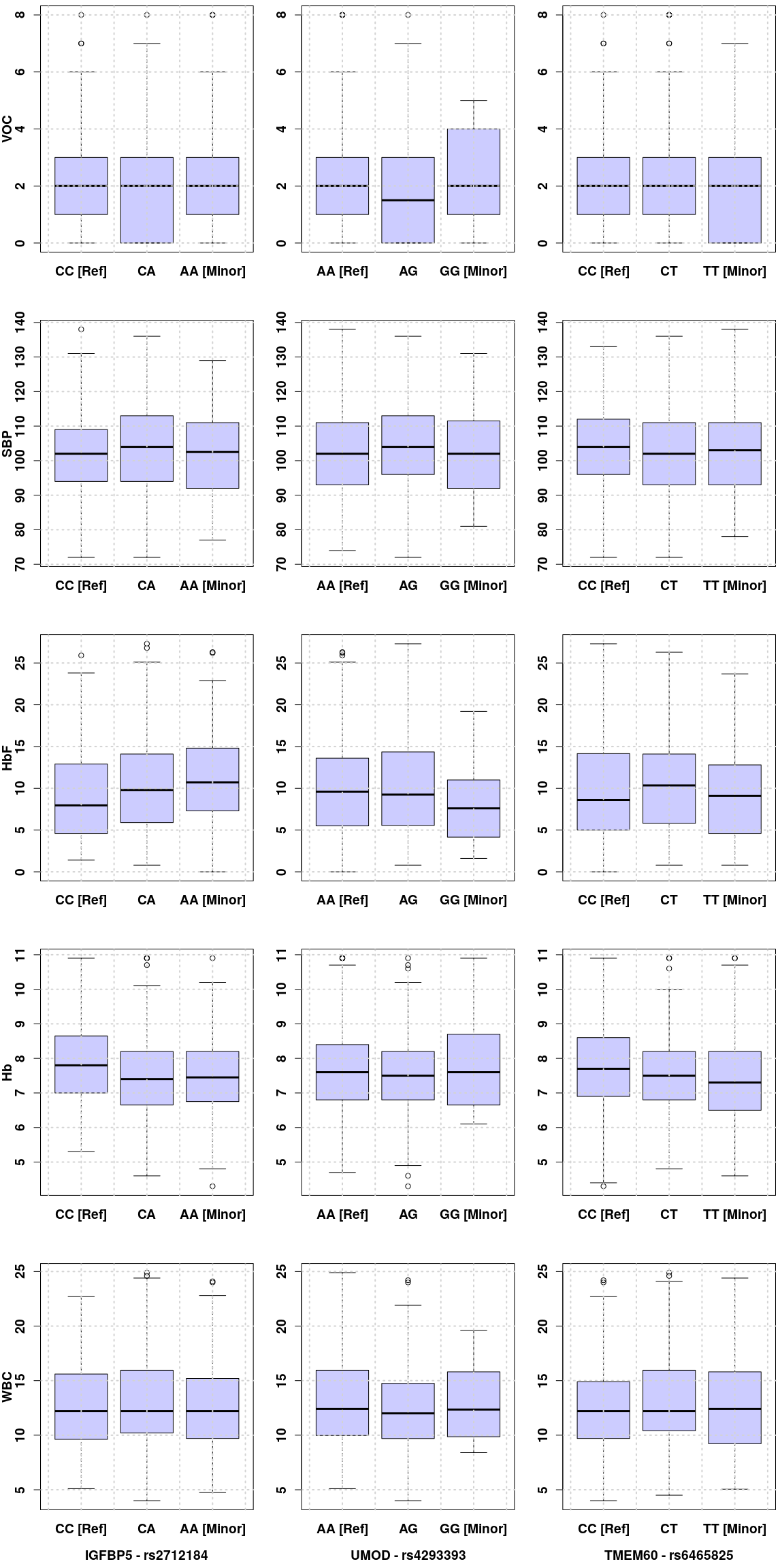


**Fig S2**: **Box plots showing variation of different significant environmental factors vs alleles in the kidney dysfunction-related variants in overall population.** The presence of the heterozygous minor allele A/G in UMOD-rs4293393 significantly decreased the number of vaso-occlusive crisis. While the minor allele A (IGFBP5-rs2712184)) and T (TMEM60-rs6465825) maintain it constant. In the heterozygous state, IGFBP5-rs2712184 and UMOD-rs4293393 variants increase systolic blood pressure while C/T in TMEM60-rs6465825 decreases this variable; the homozygous minor allele of all the genetic variants maintain SBP at its original level. For HbF, C/A, A/A and C/T increased its level while G/G and T/T tend to lower it. Hemoglobin level also decreased in the presence of C/A, A/A, A/G, C/T and T/T and increase only in the presence of G/G in UMOD. The white blood cell counts only decrease in the presence of A/G (UMOD-rs4293393).
